# Simplex and multiplex CRISPR/Cas9‐mediated knockout of grain protease inhibitors in model and commercial barley improves hydrolysis of barley and soy storage proteins

**DOI:** 10.1111/pbi.70065

**Published:** 2025-03-27

**Authors:** Michael Panting, Inger B. Holme, Giuseppe Dionisio, Henrik Brinch‐Pedersen

**Affiliations:** ^1^ Department of Agroecology – Crop Genetics and Biotechnology Aarhus University Slagelse Denmark

**Keywords:** selection‐gene‐free genome editing, protease inhibitors, model and commercial barley (*Hordeum vulgare* L.), simplex and multiplex CRISPR/Cas9, soybean storage protein degradation, barley hordein degradation

## Abstract

Anti‐nutritional factors in plant seeds diminish the utilization of nutrients in feed and food. Among these, protease inhibitors inhibit protein degradation by exogenous proteases during digestion. Through conventional and selection‐gene‐free genome editing using ovules as explants, we used simplex and multiplex CRISPR/Cas9 for studying the impact of chymotrypsin inhibitor CI‐1A, CI‐1B and CI‐2, Bowman‐Birk trypsin inhibitor, Serpin‐Z4, and barley ɑ‐amylase/subtilisin inhibitor on barley and soybean storage protein degradation. Mutants were generated in the commercial cultivar Stairway, having a high level of protease inhibition, and the barley model cultivar Golden Promise, having a lower inhibition level. In Golden Promise, all individual knockouts decreased the inhibition of the three proteases α‐chymotrypsin, trypsin and the commercial feed protease Ronozyme ProAct significantly. The triple knockout of all chymotrypsin inhibitors further decreased the inhibition of α‐chymotrypsin and Ronozyme ProAct proteases. Degradations of recombinant barley storage proteins B‐ and C‐hordeins were significantly improved following mutagenesis. In Stairway, a single knockout of CI‐1A almost compares to the effect on the proteases achieved for the triple knockout in Golden promise, uncovering CI‐1A as the major protease inhibitor in that cultivar. The Stairway mutant demonstrated significantly improved degradation of recombinant barley hordeins and in the soybean storage proteins glycinin and β‐conglycinin. The results of this study provide insights into cereal protease inhibitor genes and their negative effects on the degradation of barley storage protein and the most important plant protein from soybeans. The study suggests a future focus on plant protease inhibitors as a major target for improving feed and food protein digestibility.

## Introduction

Efficient protein digestibility is essential for a healthy diet and for reducing agriculture's impact on climate and the environment. In human diets, inefficient hydrolysis of certain plant proteins can potentially lead to celiac disease (Dahal‐Koirala *et al*., 2020). From an agricultural, climate and environmental perspective, non‐digested protein from feed contributes significantly to N leaching into the environment. The European Commission has recognized the urgent need to cut down nutrient loss by at least 50% by 2030 under the Green Deal program (The European Commission, 2020). Efficient and non‐inhibited degradation of plant‐based feed protein is vitally important for reaching this goal.

Grains from cereals contain several anti‐nutritional factors interacting with enzymes important for the degradation of phytin (phytase), non‐starch polysaccharides (xylanase) and proteases (Bekalu *et al*., 2017; Dornez *et al*., 2009; Nørgaard *et al*., 2019). While the effect of cereal xylanase inhibitor levels on animal feed digestibility is well known (Krogh Madsen *et al*., 2018), the impact of individual cereal protease inhibitors is largely undescribed. The collection of protease inhibitors is huge, and each can potentially inhibit proteases of importance in foods and feed. The inhibition of proteases can potentially also affect celiac disease management because epitope peptides are not degraded, resulting in an immune response (Dahal‐Koirala *et al*., 2020). *In planta*, endogenous grain protease inhibitors have a main function in the timing of protein hydrolysis prior to and during germination, as well as functioning as a defence against fungal‐ and pest proteases (Abd El‐latif, 2014; Pekkarinen *et al*., 2003; Pekkarinen and Jones, 2002). The latter is urgently important to keep in mind and evaluate in plants with modulated protease inhibitor levels. However, protease inhibitors are abundant in the grain, and the addition of exogenous proteases to feed and food exceeding the level of inhibitors is not considered a viable solution. Moreover, varying levels and composition of protease inhibitors between cultivars and batches of cereals affect protease activity differently, potentially leading to different protein digestibility levels in e.g. feed. In line with this, significant cultivar differences in wheat grain xylanase inhibitor levels have already proved to affect the growth and feed rate in poultry feeding studies (Krogh Madsen *et al*., 2018).

The present study provides insight into an array of six barley grain protease inhibitors that alone or in combinations may influence the digestion of barley grain proteins and the world's most important source of plant protein, soy protein (Pope *et al*., 2024; Stein *et al*., 2008; Zhang *et al*., 2013). In addition to being important food and feed proteins, the abundant soybean storage proteins glycinin and β‐conglycinin are regarded as allergenic proteins and can cause hypersensitivity (Krishnan *et al*., 2009; Wang *et al*., 2014, 2023).

The implication of barley grain serine protease inhibitors on externally added proteases was studied. These included chymotrypsin inhibitors (CI‐1A, CI‐1B and CI2), barley α‐amylase/subtilisin inhibitor (BASI) a bifunctional inhibitor belonging to the Kunitz‐type trypsin inhibitor family (Nielsen *et al*., 2004), Bowman‐Birk trypsin inhibitor (BBI) and Serpin Z4. The most abundant protease inhibitors in the barley grain are the serpin Z proteins. There are three Z proteins present in the endosperm of barley, Zx, Z4 and Z7, with Z4 being the most abundant of the three and Z4 and Z7 accounting for up to 5% of the grain protein (Evans and Hejgaard, 1999). The serpins have a dual function in the grain as both serine protease inhibitors and as storage proteins, accounting for a large portion of the grain lysine (Hejgaard *et al*., 1985). Additionally, Z proteins have been identified as a beer component improving beer foaming quality (Evans and Hejgaard, 1999).

As a potentially novel approach to increase the nutritional value of plant protein, the levels of the barley protease inhibitors were reduced by simplex and multiplex CRISPR/Cas9 mutagenesis. Finally, selected mutants were tested for their effect on the degradation of recombinant barley hordein storage proteins B and C and native soy protein.

## Results

### Protease inhibition in model and modern barley cultivars

**Figure 1:**
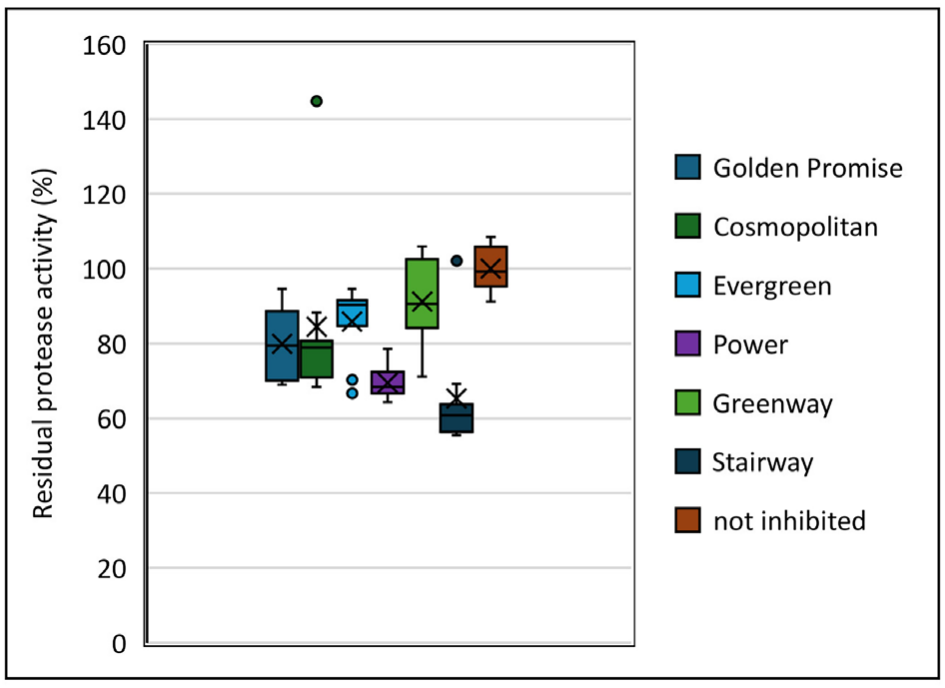
Box plots inclusive medians. Dots represent outliers and (×) is the mean value. Residual Ronozyme ProAct activity assay on elite barley cultivars and Golden Promise with 40 μL (100% grain protein fraction). The proteolysis of the AZCL‐Casein substrate is measured at 600 nm.

Golden Promise is a model barley cultivar used primarily because of its favourable tissue culture properties and is not widely grown as an agricultural crop (Bekalu *et al*., 2023). To put our study in the context of today's agriculture, we therefore tested modern barley cultivars for their inhibition of commercial Ronozyme ProAct protease with 100% grain protein extract (Figure 1). Many of the cultivars tested have a similar effect on residual protease activity as Golden Promise (70%–90%), but some differ. Greenway had the highest residual protease activity with 91% of all the cultivars tested. At the other end of the scale, Stairway allowed only 65% residual activity of the protease.

### Generation of CRISPR/Cas9 loss of function mutants

**Figure 2:**
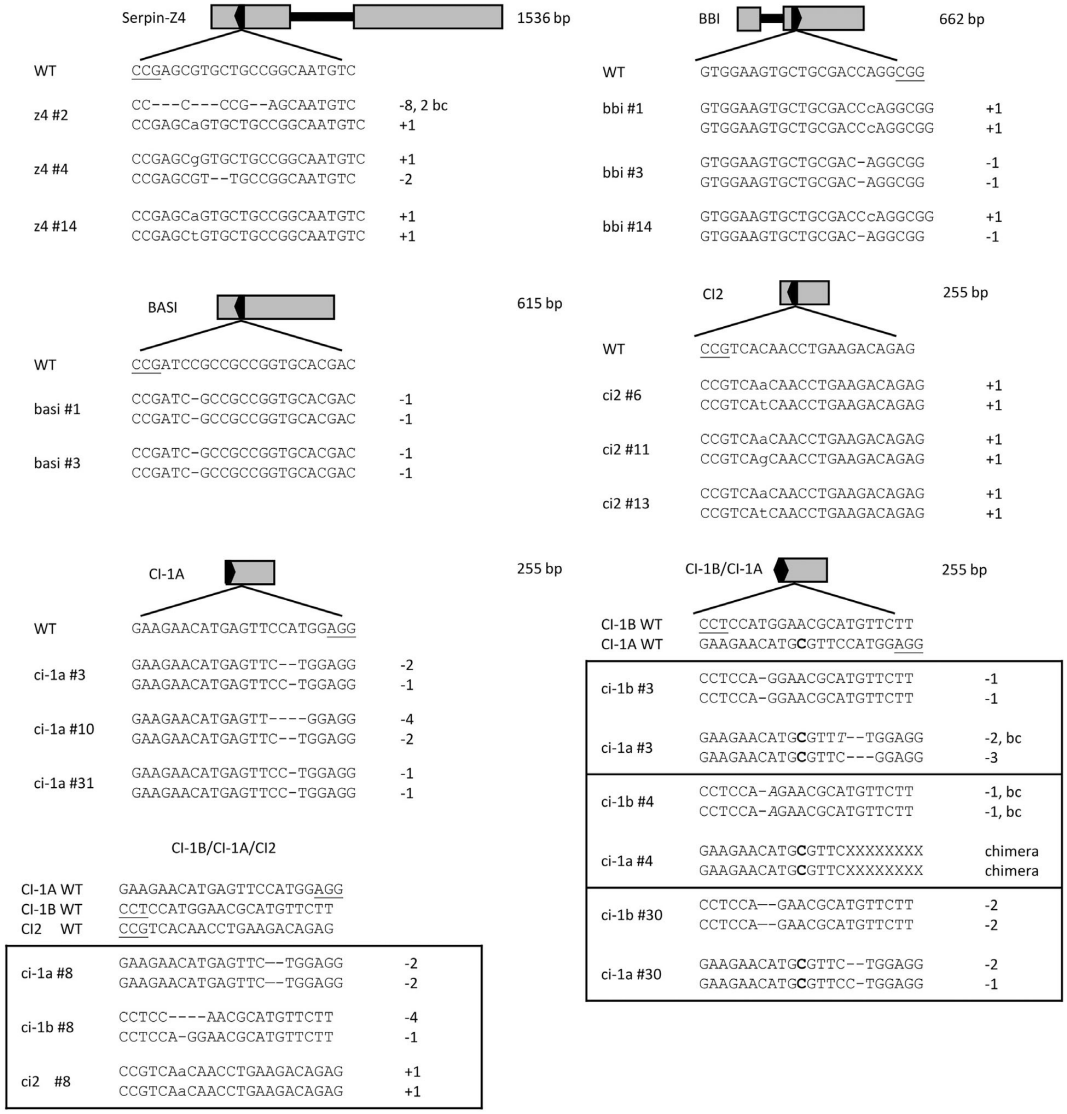
Schematic figures of protease inhibitors and mutations in Golden Promise. The wild type sequence of the protospacer sequence (WT) and both alleles of the mutant sequences selected for further analysis. Grey box, exons. Black line, introns. Black arrow, location of protospacer sequence in the gene and its orientation. The triple and double mutant lines are divided in the black boxes. Bc, base change.

Six protease inhibitor genes (*CI‐1A*, *CI‐1B*, *CI2*, *Serpin‐Z4*, *BBI* and *BASI*) expressed in the mature barley grain were identified for the cultivar Morex in the Ensembl barley genome (https://plants.ensembl.org/Hordeum_vulgare/Info/Index). Protospacers for the knockout of these genes were designed for the CRISPR/Cas9 constructs (Table 1). As the transformation target was the barley cultivar cv Golden Promise, the selected protospacer sequence of each gene was verified by sequencing the corresponding target regions in Golden Promise. The *CI‐1A* gene sequence in Stairway was also sequence‐verified as being the same as in Golden Promise. Golden Promise simplex and multiplex knockout mutant transformants were generated, and mutations were identified by sequencing (Figure 2). Transformation efficiency and regenerated plants zygosity can be seen in Tables S1 and S2. Primary mutants with homozygous or bi‐allelic out‐of‐frame mutations were selected for further studies when possible (Figure 2). Three such primary mutants could be selected for each of the *Serpin‐Z4*, *BBI*, *CI‐1A* and *CI2* genes and the double knockout of genes *CI*‐*1B* and *CI*‐*1A*. For the *BASI* gene knockout, two such lines could be selected, and only one such primary mutant was generated with the triple chymotrypsin inhibitor knockout *CI‐1B*/*CI*‐*1A*/*CI2*. Predicted amino acid translations of the mutations are shown in Figure S1. Most mutants show a non‐sense frameshift resulting in a premature stop codon. The exceptions are the bbi mutant #3 and bbi mutant #14 allele 2, showing non‐sense translation but extended sequence before a stop codon. The *CI‐1B* and *CI‐1A* double mutant plant number 3 has a three‐base deletion resulting in a single amino acid deletion in ci‐1a #3 allele 2 (Figure 2 and Figure S1). Three regenerated Golden Promise plants with wild‐type genotype were also included as controls.

**Table 1:** Protease inhibitors selected for CRISPR/Cas9 mutation

Gene name	Accession nr.	Protospacer sequence (5′‐3′)
Chymotrypsin inhibitor 1A (CI‐1A)	HORVU.MOREX.r3.1HG0012580	GAAGAACATGAGTTCCATGG**AGG**
Chymotrypsin inhibitor 1B (CI‐1B)	HORVU.MOREX.r3.1HG0012570	**CCT**CCATGGAACGCATGTTCTTC
Chymotrypsin inhibitor 2 (CI2)	HORVU.MOREX.r3.1HG0012640	**CCG**TCACAACCTGAAGACAGAG
Serpin‐Z4	HORVU.MOREX.r3.4HG0342810	**CCG**AGCGTGCTGCCGGCAATGTC
Bowman‐Birk trypsin inhibitor (BBI)	HORVU.MOREX.r3.3HG0222410	GTGGAAGTGCTGCGACCAGG**CGG**
Barley α‐amylase/subtilisin inh. (BASI)	HORVU.MOREX.r3.2HG0183360	**CCG**ATCCGCCGCCGGTGCACGAC

Their accession number and protospacer sequence. PAM site as bolded.

Two of the chymotrypsin inhibitors (*CI‐1A* and *CI‐1B*) have a high gene sequence homology of 93.1% (Figure S2a). As a result of this, the designed protospacers for these two genes have a single nucleotide difference (A/C) at the tenth nucleotide from the PAM site (Figure S2b). This SNP is at the border of what is designated as the seed region of the protospacer, normally defined as being between the 8^th^ and 10^th^ nucleotides proximal to the PAM site (Soyars *et al*., 2018). By using the border‐lining SNP, we sought to generate both single and double mutants using a single guide with the protospacer designed for the *CI‐1A* gene or the protospacer designed for the *CI‐1B* gene in a simplex system (Table 1). The CRISPR/Cas9 construct with the protospacer designed for the *CI*‐*1A* only induced mutations in the *CI*‐*1A* gene but did not induce any mutation in the *CI‐1B* target gene, being wild type in all plants (Figure S2c). On the other hand, the CRISPR/Cas9 construct with the protospacer designed for the *CI‐1B* gene induced mutations in both the *CI‐1B* and the *CI‐1A* gene in all transformants screened (Figure S2c). We therefore managed to obtain single knockout mutants of chymotrypsin inhibitor 1A (ci‐1a) and double knockout mutants of chymotrypsin inhibitors 1A and 1B (ci‐1b/1a).

The three genes *CI‐1A*, *CI‐1B and CI2* are chymotrypsin inhibitors, belonging to the Potato type I inhibitor family. Their identical molecular function as serine‐type endopeptidase inhibitors (gene ontology, GO: 0004867) results in an overlap of inhibitory functions, as described previously (Greagg *et al*., 1994). To investigate the additive effect of these three CI genes, we generated a multiplex triple chymotrypsin inhibitor knockout (ci‐1a/1b/2).

### Effect of mutations in barley on external protease activity

**Figure 3:**
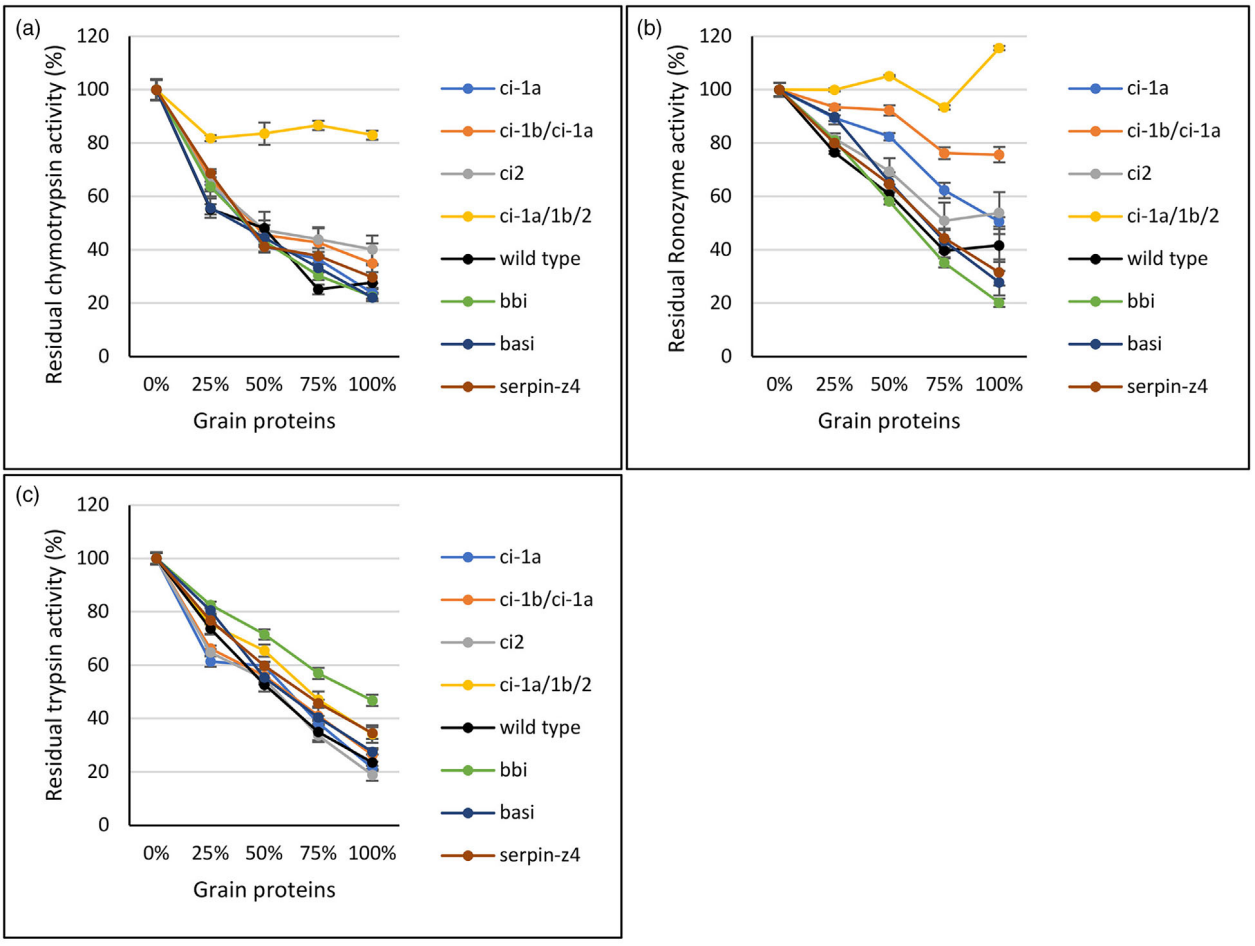
Residual protease activity assay. Extracted water‐soluble grain protein fractions from mutants or wild type mature grains are mixed with a protease to measure the inhibitory effect of protease inhibitors in the grain. Samples from each plant are run in a technical triplicate. The wild type ci‐1b/1a double mutant and all single mutants have three biological replicates (*n* = 9), except BASI with two mutant plants (*n* = 6). Three separate protein extractions from the ci‐1a/1b/2 triple mutant were used (*n* = 9). The proteolysis of the AZCL‐Casein substrate is measured at 600 nm. (a) Residual protease activity of α‐chymotrypsin. (b) Residual protease activity of Ronozyme ProAct. (c) Residual protease activity of trypsin.

Mutant and wild type barley grain proteins were assayed for their inhibition of the three different serine proteases: α‐chymotrypsin (C4129, Sigma‐Aldrich), trypsin (T8003, Sigma‐Aldrich) and the commercial feed additive protease Ronozyme ProAct, a subtilisin with mainly chymotrypsin activity (Novozymes, Denmark). Mature grains were milled and different amounts of water‐soluble protein extracts from the flour (0%, 25%, 50%, 75% and 100%) were mixed with the proteases. These extracts will be referred to as grain proteins. The residual activity of α‐chymotrypsin showed that wild type grain protein at 25% reduced the protease activity to 55% (Figure 3a). The activity decreased further to 25% and 28% when 75% and 100% grain proteins were added, respectively. With the additions of 75% wild type grain protein, all mutants performed significantly better than the Golden Promise wild type, except for the bbi mutants. At 100% grain protein, only the ci‐1a/1b/2 triple mutant performed significantly better than the wild type. The residual protease activity of the ci‐1a/1b/2 triple mutant was at all grain protein fractions significantly higher in the mutated line than in the wild type. The residual activity of the ci‐1a/1b/2 triple mutant stayed between 81.8% and 86.7%, regardless of the amount of grain protein added to the reaction. At 25% grain protein, the single ci‐1a mutants and ci‐1b/1a double mutants showed with 65% and 67% residual activity, respectively, a significantly increased residual protease activity as compared to the wild type. The ci2 mutants had the highest residual activity of the single mutants, ranging from 65% activity with 25% grain protein to 40% with 100% grain protein (Figure 3a).

Next, the residual activity of Ronozyme ProAct protease was measured (Figure 3b). The inhibition of Ronozyme ProAct by the wild type grain protein extract was less than the inhibition of α‐chymotrypsin, ranging from 76% activity at 25% grain protein and having the lowest activity at 75% grain protein with 40% activity. Grain proteins from bbi and basi mutants both had a significantly higher residual activity than the wild type when 25% grain protein was added, displaying a residual activity of 81% and 90%, respectively. However, at 100% grain protein, the bbi mutant had significantly lower activity than the wild type, with only 20% residual activity, while the basi mutant showed 28% residual activity. The knockout of chymotrypsin inhibitors had a great effect on the residual activity of Ronozyme ProAct (Figure 3b). With 25% grain protein, all four knockout combinations had a significantly positive effect on Ronozyme ProAct activity. The ci2 mutant displayed a minor increase with 82% residual activity. Ci‐1a, ci‐1b/1a double mutant, and ci‐1a/1b/2 triple mutant had 89%, 93% and 100% residual activity, respectively. With 50%, 75% and 100% grain protein, the ci2 mutant still had the lowest residual activity at 69%, 51% and 54%, respectively. The ci‐1a mutant showed significantly higher activity with 50% and 75% grain protein added (82% and 62% activity left). At 100% grain protein, the Ronozyme ProAct activity was reduced to 51%. The ci‐1b/1a double mutant showed a strong positive effect on the residual activity compared to the wild type at all levels of added grain protein, 92%, 76% and 75.7% residual activity when 50%, 75% and 100% grain proteins were added, respectively. Again, the ci‐1a/1b/2 triple mutant showed the highest effect on the residual protease activity (Figure 3a,b). Interestingly, at both 50% and 100% albumin, the activity exceeded the starting level of pure Ronozyme ProAct protease (0% grain protein, 100% activity). Surprisingly, the highest activity (116%) was found with the highest amount of grain protein added. Serpin‐Z4 showed almost the same residual activity as the wild type at all concentrations of grain protein (Figure 3b).

The third protease measured for its residual activity with grain protein fractions was bovine trypsin (Figure 3c). As expected, there was less effect of the knockout of chymotrypsin inhibitors. Indeed, at 25% grain protein, ci‐1a, ci‐1b/1a and ci2 had lower activity than the wild type (61%, 66%, 65% and 74%, residual activity detected respectively). The residual activity level of the ci‐1a/1b/2 triple mutant was like the wild type (75%) with 25% grain protein. At 50%, 75% and 100% grain protein, ci‐1a/1b/2 showed higher residual activity than the wild type with 65%, 47% and 34% residual trypsin activity in the triple mutant as compared to 53%, 35% and 23% in the wild type. The basi mutant retained a higher residual activity at 25% grain protein, but not when adding more grain protein. The serpin‐z4 mutant performed better at 50% grain protein with residual trypsin activity levels at 60%, 46% and 35% with 50%, 75% and 100% grain proteins, respectively. The bbi knockout mutant displayed the highest effect on the residual activity of trypsin, being significantly higher than the wild type at all grain protein fractions. The residual trypsin activity was 83% at 25% grain protein, 71% at 50% grain protein and 47% at 100% grain protein.

### Native protein degradation

**Figure 4:**
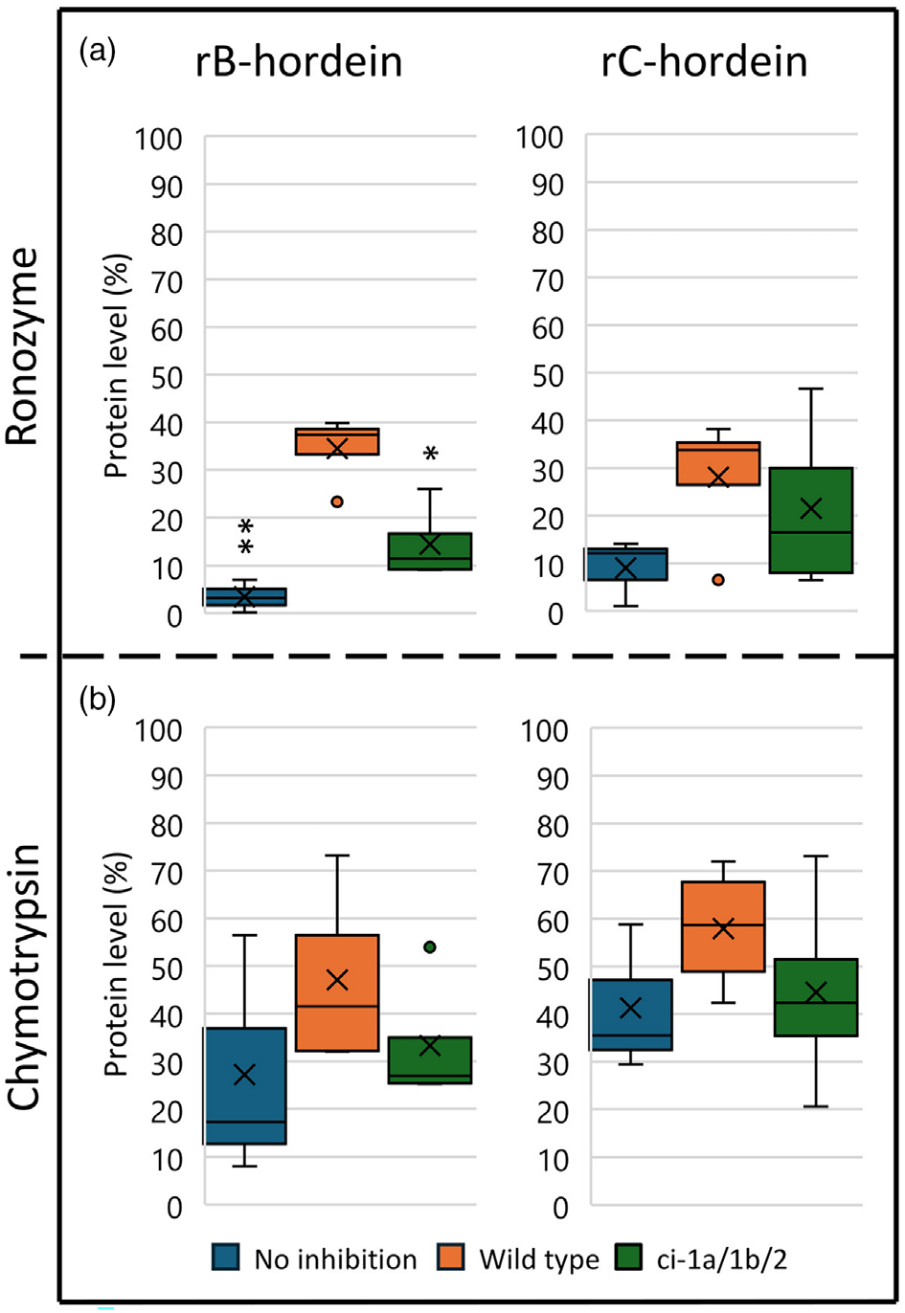
Box plots inclusive medians of remaining substrate protein level (%). The band intensities within an area on SDS‐PAGE gels were estimated. (×) is the mean value and the dots are outliers. Protein levels were calculated from the protein level of a control with only substrate (20 μg storage protein) given as 100% protein level. The Golden Promise ci‐1a/1b/2 triple mutant and three wild type regenerated plants water‐soluble grain protein fractions were incubated with either recombinant B‐ or C‐hordein and tested for their inhibition of Ronozyme ProAct and α‐chymotrypsin (a and b). A mix of three independent grain protein extractions from the triple mutant was pooled before incubation and loading on the gel. The same was done for the three wild types. Four independent runs were made and used for protein level calculations (*n* = 4). Samples without any grain proteins were included for all experiments (No inhibition, *n* = 3 in all experiments). Significant differences were calculated by two‐tailed Student's *t*‐test. *P < 0.05, **P < 0.01.

The triple chymotrypsin mutant ci‐1a/1b/2 was the best performing mutant in the residual protease assay using α‐chymotrypsin or Ronozyme ProAct (Figure 4a,b). This mutant was therefore selected for a recombinant protein degradation assay of storage barley proteins. The storage proteins B‐ and C‐hordein were expressed in *E. coli* and purified. The recombinant storage proteins were mixed with the water‐soluble grain protein fraction of the triple mutant or wild type. Controls having no protease and grain protein (pure hordein) were used for calculating the relative degradation of hordeins, measured as the adjusted volume of band intensities in per cent (protein level, %). A control with only protease and hordein (no inhibition) was included to see how much recombinant hordein would be degraded without inhibition (Figure 4a,b). With Ronozyme ProAct protease and the non‐inhibited control, almost all the rB‐ and rC‐hordein substrates were degraded (Figure 4a). The wild type grain inhibited the degradation of rB‐hordein using Ronozyme ProAct with a mean residual substrate protein level of 34.5%. Both the ci‐1a/1b/2 triple mutant and no inhibition samples inhibited degradation significantly less than the wild type, with substrate protein levels at 14.5% and 3.4%, respectively (Figure 4a). With the rC‐hordein Ronozyme ProAct degradation (Figure 4a) and the degradation of both recombinant proteins with α‐chymotrypsin (Figure 3b), there is a tendency for the wild type samples to have a higher inhibition. This is the case for both the non‐inhibited sample and ci‐1a/1b/2 triple mutant samples.

### CRISPR/Cas9 mutagenesis of the modern barley cultivar Stairway

**Figure 5:**
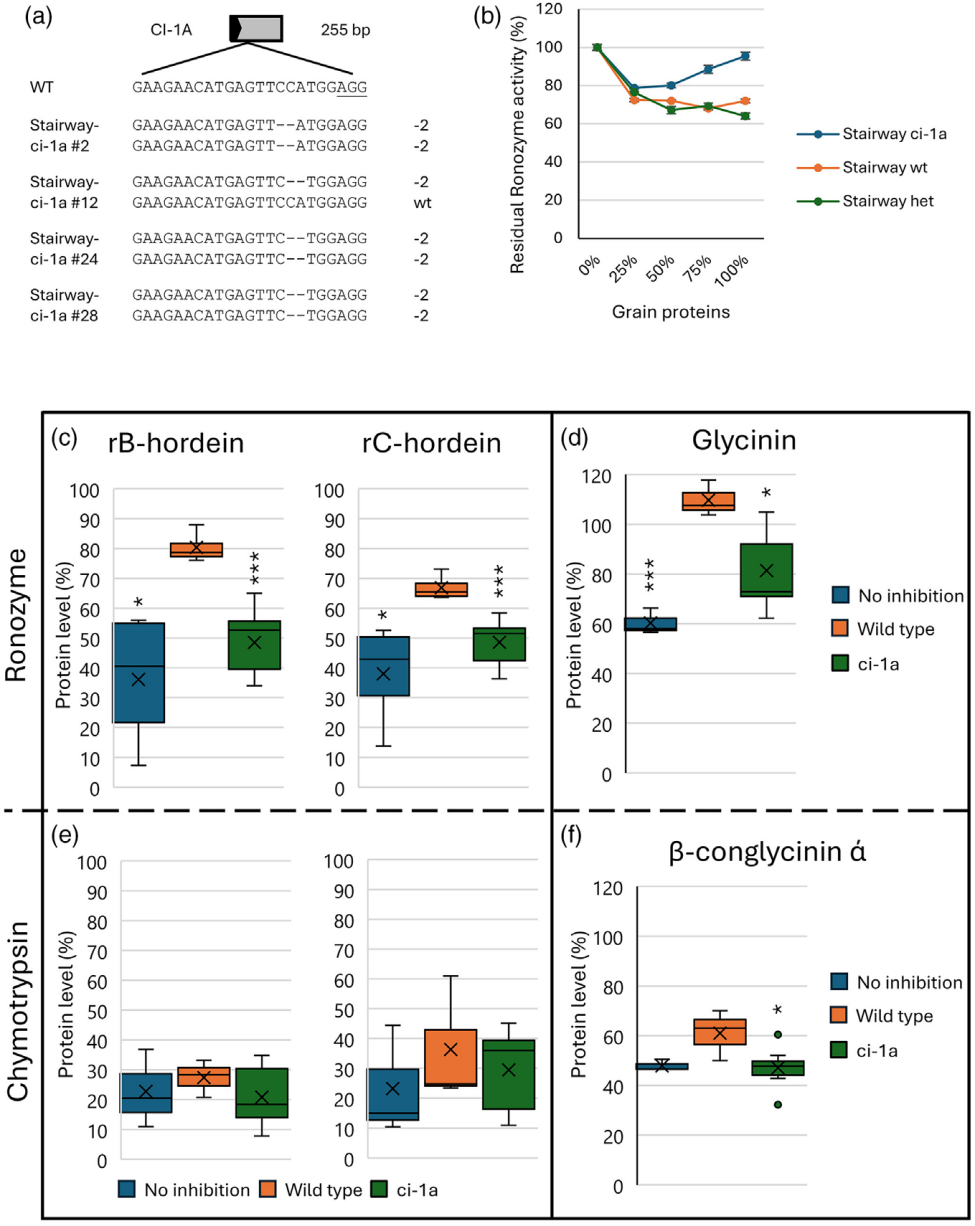
Stairway cultivar mutants. (a) Schematic figure of CI‐1A protease inhibitor mutations in T_1_ generation Stairway ci‐1a mutants. The described lines are used in all subsequent experiments. Grey box, exons. Black arrow, location of protospacer sequence in the gene and its orientation. (b) Residual protease activity of Ronozyme in the T_1_ generation of Stairway mutants, Stairway wild type, and Stairway heterozygous mutants. The proteolysis of the AZCL‐Casein substrate is measured at 600 nm. Protease inhibition on 40 μg recombinant rB‐ or rC‐hordein was tested with the water‐soluble grain proteins from the Stairway ci‐1a mutants and the wild type and run on SDS‐PAGE gels (c, Ronozyme ProAct and e, α‐chymotrypsin). The three T_1_ mutant siblings were run independently three times and the protein levels were pooled (*n* = 9). The wild type T_1_ sibling was run three times (*n* = 3). Inhibition of Ronozyme ProAct and α‐chymotrypsin by Stairway ci‐1a mutants and wild type grain proteins on degradation of soy storage proteins glycinin (d) and β‐conglycinin ά (f). Samples were run like the hordein experiment with *n* = 9 for the Stairway ci‐1a mutants and *n* = 3 for the wild type. Samples without any grain proteins were included for all experiments (No inhibition, *n* = 3 in all experiments). Significant differences were calculated by two‐tailed Student's *t*‐test. *P < 0.05, ***P < 0.001.

Having the highest potential for improvement (Figure 1), the commercial barley cultivar Stairway was selected for targeted mutagenesis of protease inhibitors. Stairway was mutated in the *CI‐1A* gene through *Agrobacterium*‐mediated transformation of the ovule. The procedure, including embryo development from the zygote within the ovule and the subsequent plant regeneration from the embryo, was performed without a selection agent in the culture media used. One mutant plant was regenerated (Figure S3a). The sequencing of the target site in the *CI‐1A* gene of the T_0_ mutant revealed a bi‐allelic mutation at the target site, but sequencing of the T_1_ progeny showed that it was chimeric, with wild type, heterozygous and homozygous mutant offspring (Figure S3b,c). In the T_1_ offspring, we identified one wild type plant, three homozygous ci‐1a mutant plants and two heterozygous plants (Figure 5a). One of these (number 11) could only be verified in the PCR/RE assay, while no sequence could be obtained. The amino acid sequence of the mutations shows a premature stop codon shortly after the mutations (Figure S3d). These were tested for the residual protease activity assay using Ronozyme ProAct, because this protease showed the highest variation in Golden Promise (Figure 3b). The wild type and heterozygous mutant plants show very limited variation in the residual protease activity (Figure 5b). On the other hand, the Stairway ci‐a1 homozygous mutants showed significantly increased residual activity from 50% grain protein levels and up. Interestingly, residual protease activity increased from approximately 80%–96% when 50%–100% grain protein was added. The wild type plant had 72% residual activity with 100% grain protein (Figure 5b).

Next, degradation of recombinant barley storage proteins rB‐ and rC‐hordein with either Ronozyme ProAct or α‐chymotrypsin proteases was studied (Figure 5c,e). Grain protein fractions were added from wild type or the Stairway ci‐1a homozygous T_1_ mutants (ci‐1a mutants). The Ronozyme ProAct protease degraded significantly more rB‐ and rC‐hordein in the ci‐1a mutant lines compared to the wild type line (Figure 5c).

A‐Chymotrypsin was able to degrade most of both rB‐ and rC‐hordein in all samples. The highest remaining substrate protein levels were in the wild type with a mean of 27.4% and 36.6%, respectively (Figure 5e). To compare, the ci‐1a mutants had a mean remaining substrate protein level of rB‐ and rC‐hordein at 20.8% and 29.5%, respectively.

### Soy storage protein degradation

One of the most important plant protein sources is soy proteins. The two most abundant storage proteins in soy are the water‐soluble glycinin (11S glycinin) and β‐conglycinin (7S glycinin) accounting for 70%–80% of total protein content in the mature grain (Singh *et al*., 2015). The Stairway ci‐1a mutants were studied for their potential to stimulate improved soy protein degradation (Figure 5d,f). The substrate protein level (%) from the SDS‐PAGE gel of the degradation assay was estimated, relative to the pure soy samples (100%). The degradation of glycinin by Ronozyme ProAct was significantly higher in the no inhibition sample and ci‐1a mutants sample compared to the wild type (Figure 5d). It appears that the grain protein fraction has protein at the same size as glycinin (~28 kDa), giving a protein level of 109.7%, which is higher than the pure soy sample. Degradation of the β‐conglycinin bands around 72 kDa (type ά) by α‐chymotrypsin was significantly higher in the ci‐1a mutants than the wild type (Figure 5f). The remaining substrate protein level was the same in the ci‐1a mutants as for the no inhibition sample (47.1% and 47.8%, respectively).

In order to determine if there were any negative phenotypical impacts of the mutants, we evaluated the agronomic traits spike length, spike number and TKW in the Golden Promise ci‐1a/1b/2 triple mutant and the Stairway ci‐1a mutant. No significant differences were detected in any of the parameters (Figure S4a). Additionally, the spike morphology remained unchanged for all studied mutants (Figure S4b).

## Discussion

Grain protease inhibitors represent significant anti‐nutritional factors, reducing the nutritional value of grain protein in food and feed. Inefficient protein digestibility has significant implications for the health value of a diet and on how much non‐digested nitrogen is secreted into the environment via the manure. Our study aimed to identify important protease inhibitor genes that function as negative regulators of the proteolysis of the major storage proteins from barley and soybean. By using simplex and multiplex CRISPR/Cas9 mutagenesis, we aimed to increase the digestibility of barley grain proteins and soy protein by removing serine protease inhibitors in the grain (Adeola and Cowieson, 2011; Canibe and Jensen, 2003; Christensen *et al*., 2014; Hejgaard and Boisen, 1980; Holme *et al*., 2012a; Nørgaard *et al*., 2019). Our research included studies in the model barley cultivar Golden Promise and in modern commercial barley cultivars. Six protease inhibitor candidates were selected and single knockout mutants were obtained for Serpin‐Z4, BASI, BBI, CI‐1A and CI2. In addition, CI‐1A/CI‐1B double mutants and a CI‐1A/CI‐1B/CI2 triple mutant plant were obtained by multiplex CRISPR/Cas9 (Figure 2). Due to high sequence homology between the *CI‐1A* and *CI‐1B* genes and only a single SNP in the protospacer sequences, we did not obtain a single CI‐1B mutant. Interestingly, the mutation rate differed between the two guides at their alternative target site, with the *CI‐1B* guide generating mutations in the *CI‐1A* gene, while the *CI‐1A* guide did not generate any mutations at the alternative *CI‐1B* target site. Further studies are required to uncover the background for this difference.

With the exception of Golden Promise and a few other cultivars, barley is known to be recalcitrant in tissue culture, and hence also gene editing (Bekalu *et al*., 2023). However, Golden Promise is of only limited relevance for current agriculture, and we therefore decided to involve modern and relevant cultivars in our study. Five different modern barley cultivars and Golden Promise were tested *in vitro* for their inhibitory effect of the Ronozyme ProAct feed protease (Figure 1). The inhibition varied significantly between the cultivars. The Greenway cultivar caused the least inhibition, maintaining 91.1% protease activity, while the cultivar Stairway showed the highest inhibition, leaving only 65.2% activity. The varying levels of protease inhibition between cultivars are in line with cultivar differences seen for inhibition of the nutritionally important enzymes xylanase and phytase (Bekalu *et al*., 2017; Krogh Madsen *et al*., 2018).

By using the barley ovule as the starting explant and no selective agent in the media used during the culture of the *Agrobacterium* infected tissue, a mutation in the *CI‐1A* gene was introduced by CRISPR/Cas9 in Stairway, the cultivar with the highest endogenous protease inhibition. Barley ovules have previously been demonstrated as a source for cultivar‐independent explants for genetic transformation (Holme *et al*., 2008). However, the current study is the first demonstration of the barley ovule as an explant for gene editing in barley. Moreover, the gene editing was done in the commercial cultivar Stairway, which in our lab has been impossible to approach for transformation and gene editing via the standard procedure using immature embryos as the starting material. The whole procedure was carried out without the use of a selective agent. To our knowledge, this is the first example of gene editing in commercial barley not involving a selective agent.

From the T_0_ chimeric Stairway mutant, we selected three homozygous mutants, two heterozygous mutants and one wild type T_1_ progeny. The level of residual activity of Ronozyme ProAct after assaying with grain water‐soluble protein from the homozygous ci‐1a mutants was significantly increased when compared to the heterozygous and wild type plants. The activity even increased with increased grain protein (Figure 5b). A similar tendency was observed for the ci‐1a/1b/2 triple mutant (Figure 3b), but the mechanism behind this needs to be studied in detail. The single gene knockout of *CI‐1A* in Stairway and Golden Promise had different effects on the residual activity of Ronozyme ProAct. The Stairway ci‐1a mutants had a 95.5% residual activity compared to 50.5% residual activity in the ci‐1a Golden Promise mutants when assayed with 100% grain protein. The result demonstrates how the level of a single protease inhibitor can vary between cultivars. The current study represents a conceptual study but does also indicate that the level of individual protease inhibitor contributions in a cultivar must be taken into account in the planning of a breeding strategy supporting a better proteolysis of storage proteins.

In Golden Promise, there is a clear increase in residual activity of α‐chymotrypsin and Ronozyme ProAct protease when knocking out all three chymotrypsin inhibitor genes compared to knocking out only one or two genes (Figure 3b). The ci‐1a/1b/2 triple mutant shows an activity higher than 100% when assayed with 100% grain protein extracts, which is more than the protease without added grain protein. One explanation could be that endogenous proteases are present in the crude water‐soluble fraction of barley grain proteins, affecting the degradation of the substrate.

As expected, as a trypsin inhibitor, the *BBI* knockout had the highest effect on the level of residual activity of trypsin after mixing with grain protein. The *bbi* mutant showed the highest increase in protease activity of all mutants from 25% to 100% added grain protein. Knockout of the *Serpin‐Z4* gene encoding the very abundant Serpin‐Z4 protein also showed a higher residual trypsin activity from 50% grain protein and above (Figure 3c).

In barley, the most abundant grain storage protein is B‐hordein, accounting for 70%–90% of the total hordeins, followed by C‐hordeins ranging from 10% to 30% of the total hordeins (Shewry *et al*., 1985; Tanner *et al*., 2019). In this study, improved degradation of recombinant barley hordeins by Ronozyme ProAct or α‐chymotrypsin was seen in the Golden Promise chymotrypsin triple mutant. The recombinant rB‐hordein was significantly more degraded by Ronozyme ProAct in the presence of grain protein extracts from ci‐1a/1b/2 triple mutant than extracts from the wild type (Figure 4a). Also, the extracts from the Stairway ci‐1a mutants led to significantly improved digestibility by Ronozyme ProAct of recombinant rB and rC hordeins as compared to extracts from the wild type (Figure 5c). The Stairway ci‐1a mutants were also studied for their effect on the degradation of soybean storage proteins (Zhang *et al*., 2013). Also, here we found that the mutants performed better, with higher degradation by Ronozyme ProAct or α‐chymotrypsin of glycinin and β‐conglycinin compared to the wild type (Figure 5d,f). Our results provide the first insight into a novel way of mutating barley that potentially can lead to increased digestibility of important storage proteins from barley and soybean and suggest that future efforts could be directed towards understanding how these changes could be utilized in the context of food and feed.

In conclusion, the findings of this study could prove to have a positive nutritional impact on barley used as feed or food and perhaps also on malting quality. It could also result in less or better utilization of protein sources, i.e. soy or protease additives in feed.

## Experimental procedures

### Vector construction

The vector system used for CRISPR/Cas9‐induced mutations relies on the destination vector pANIC6A (Mann *et al*., 2012). In this system, two entry vectors are used: the pJG85 (addgene #89281) entry vector for insertion of the specific synthetic guide sequences (sgRNAs) and pJG80 (addgene #89282) containing the Cas9 gene, codon optimized for wheat (Gil‐Humanes *et al*., 2017). The ligation of sgRNA sequences into pJG85 and subsequent LR clonase reaction with the two entry vectors and the destination vector to assemble the final expression vector has previously been described (Panting *et al*., 2021). The sgRNA oligo sequences for all six genes can be seen on Table S3. Linearization of the entry vector using Esp3I leaves a 4 bp overhang as well as removes the first base in the scaffold RNA sequence that needs to be reconstituted in the sgRNA oligos.

The final expression vectors were transformed into *Agrobacterium tumefaciens* strain AGL0 using the freeze/thaw method. The transformed *Agrobacterium* cells were grown at 28 °C in solid or liquid LB media containing 25 μg/mL rifampicin and 50 μg/mL kanamycin antibiotics for selection.

### Introducing CRISPR/Cas9 constructs in barley immature embryos and zygotes

The donor plants (cv. Golden promise and cv. Stairway) used for transformation were grown in a growth chamber with a 16 h light period with 350 μ E m^−2^ s^−1^ and 15 and 10 °C day and night temperatures, respectively. Stable transformation of cv. Golden Promise was done by *Agrobacterium*‐mediated transformation of immature embryos. Twelve‐ to fourteen‐day‐old embryos were isolated and transformed as previously described (Holme *et al*., 2017). Briefly, the axis was cut away from the scutellum using a scalpel dipped in an overnight *Agrobacterium* culture containing no antibiotics. When generating the triple chymotrypsin inhibitor mutant, the overnight *Agrobacterium* cultures for each of the three genes were mixed 1:1:1 just prior to immature embryo transformation. The selection agent hygromycin was used at a concentration of 50 mg/L in all media except the co‐cultivation medium, which contained no selection agent.

*Agrobacterium*‐mediated transformation of cv. Stairway used the zygote within the ovules as the transformation target (Holme *et al*., 2008, 2012b). Hand‐pollination of emasculated spikes was performed 1 h before ovule isolation to ensure that the zygote within the ovules was just formed. Isolated ovules were subsequently infected with an overnight *Agrobacterium* culture containing no antibiotics. A fine needle (0.4 mm × 19 mm) dipped in the overnight culture was used to puncture the ovule embryo sac and release *Agrobacterium* into the sac containing the zygote. Subsequently, a plant was regenerated as described (Holme *et al*., 2008). The entire procedure was performed without a selection agent in any of the culture media.

### Mutant screening of genotyping

Approximately 10 cm young leaf pieces were cut and immediately frozen in liquid nitrogen in 2 mL tubes containing two 2 mm glass beads. The frozen leaves were crushed in a FastPrep‐24 5G homogenizer (MP Biomedicals) at speed 6 for 10 s. DNA was extracted using the phenol/chloroform method.

Regenerated plants were initially screened by amplifying the hygromycin gene (Table S4), using Herculase II Fusion DNA polymerase (Agilent).

Hygromycin‐positive plants were genotyped by amplifying the target region corresponding to the target in the construct also using Herculase II Fusion DNA polymerase (Agilent). The PCR products were either sequenced using the PCR amplification primers or TOPO cloned (Zero Blunt™ TOPO™, Invitrogen™), with 6–8 clones sequenced. Primers used for the different CRISPR/Cas9 targets and the PCR product sizes can be seen in Table S4.

### Protease inhibition assay

The protease inhibition assay was adapted from (Nørgaard *et al*., 2019). The inhibition effect of barley grain extracted proteins was measured for three different proteases, bovine trypsin (T8003, Sigma‐Aldrich), bovine α‐chymotrypsin (C4129, Sigma‐Aldrich) and Ronozyme ProAct (Novozymes). Samples from all lines described in Figure 1 were used in the experiment. Each line was run in technical triplicates. The crude water‐soluble albumin protein fraction (grain protein) of mature barley grains was extracted by mixing 250 mg barley flour with 2 mL of 0.1 M acetate, pH 5, as extraction buffer. The samples were shaken for 1 h in a horizontal shaker and centrifuged at 3600 × ***g*** for 5 min at 4 °C. The water‐soluble grain proteins in the supernatant were transferred to new 1.5 mL Eppendorf tubes and kept on ice until use or at 4 °C for short‐term storage. Trypsin and α‐chymotrypsin were dissolved in 0.1 M HCl (1 mg/mL) just prior to use, and Ronozyme ProAct was diluted 1:100 before use. The assay is based on the cleavage and release of the chromogenic compound (azurine) from its crosslink to casein (AZCL‐casein, Protazyme AX, Megazyme, Ireland). The AZCL‐casein substrate was prepared by dissolving two tablets in 10 mL of the reaction buffer, keeping the substrate on a magnetic stirrer all the time. The reaction buffer for Ronozyme ProAct was 0.1 M Tris/HCl, pH 8. The reaction buffer for trypsin and α‐chymotrypsin was 0.1 M Tris/HCl, pH 7.8 with 10 mM CaCl_2_.

The assay was performed with different amounts of soluble grain protein fraction, i.e., 40 μL (100%), 30 μL (75%), 20 μL (50%), 10 μL (25%) and 0 μL (0%). Extraction buffer was added to the 75%, 50% and 25% grain protein fractions for a total of 40 μL. The protease activity was also measured without any grain proteins (non‐inhibited sample, 0%). A blank control with only buffers was also included. The grain protein fractions were pre‐incubated in a 2 mL Eppendorf tube with 10 μL of protease for 5 min at room temperature. The samples were transferred to a 37 °C heating block, and 300 μL AZCL‐casein buffered substrate was added. The reaction was incubated for 5 min with Ronozyme ProAct, 15 min with trypsin, and 30 min with α‐chymotrypsin. The reaction was stopped by adding 150 μL 1.5 M HCl. The samples were centrifuged at 20 000 × ***g*** for 2 min, and 200 μL of supernatant was transferred in triplicate to a 96‐well plate. The absorbance was measured at 600 nm using an Epoch microplate spectrophotometer (BioTek AG, Germany).

The pathlength‐corrected absorbance was subtracted from the absorbance of the blank sample. The percentage of inhibition by the grain proteins was calculated by % protease inhibition = non‐inhibited sample – sample/non‐inhibited sample × 100. The remaining activity was calculated by subtracting this from 100%.

### Barley storage protein degradation assay

Recombinant barley storage proteins C‐hordein and B‐hordein (rC‐hordein and rB‐hordein, respectively) were expressed and purified from *E. coli* shuffle T7 cells (New England Biolabs) as described previously (Rosenkilde *et al*., 2014). Briefly, the expression of recombinant protein was induced by adding IPTG (final concentration of 0.5 mM) to an overnight grown 200 mL culture and left for 6 h at 25 °C. The cultures were centrifuged for 15 min at max speed. Retrieval of the recombinant protein was done using a sonication with an extraction buffer containing 0.1 M Tris/HCl pH 8.0 plus 1 mM PMSF with lysozyme. The sonicated homogenate was centrifuged at 4000 × ***g*** for 10 min, and the insoluble pellet was washed twice before further usage. The rC‐hordein was purified from the soluble fraction after sonication and centrifugation using a 20 mL Ni/NTA column (Qiagen), following the manufacturer instructions. The rB‐hordein was extracted in denaturing conditions with 8 M urea in the extraction buffer from the washed insoluble pellet. The urea‐solubilized pellet was passed through a 20 mL Ni/NTA column (Qiagen), washed with a washing solution containing 20 mM imidazole, and eluted in 250 mM imidazole pH 8.0 containing 4 M urea. The concentration of purified recombinant hordeins was measured using the Bradford method (Bradford, 1976).

The grain protein fraction from the three wild type transformants was mixed 1:1:1, as well as the three separately extracted fractions from the ci‐1a/1b/2 triple mutant. The storage protein degradation assay was done by mixing 60 μL reaction buffer (see above) with 1 μL α‐chymotrypsin (1 mg/mL) or 1:100 dilution of Ronozyme ProAct and 6 μL of grain protein fraction from the ci‐1a/1b/2 triple mutant or wild type. Samples were incubated for 5 min at room temperature and put on ice. Six μL recombinant hordein (20 μg total) was added, and the samples were incubated at 37 °C for 5 min. The reaction was stopped by putting the samples back on ice, adding 73 μL 2 × SDS‐PAGE loading buffer, and boiling the samples for 5 min. The samples were spun down at 20 000 × ***g*** for 1 min and kept on ice until loading on the gel. Ten μL of each sample was run on Nupage 4%–12% Bis‐Tris gels (Life‐Technologies). A sample only containing the recombinant rB‐ or rC‐hordein (pure hordein) was used to calculate a relative volume intensity, assuming each of those to be 100%. A control sample without grain protein (no inhibition) was included.

The degradation assay with the Stairway T_1_ mutants was as described above, but with double the amount of recombinant hordeins in the reaction (40 μg total) and the Ronozyme ProAct protease was diluted 10 times more to 1:1000.

The relative volume intensities were calculated at the band of rB‐ or rC‐hordein using the Volume tool in the Image Lab 6.1 software (Bio‐Rad). The relative volume intensities (%) were calculated based on the pure hordein samples being 100%. This will be termed as protein level (%).

### Soy protein extraction

Water‐soluble soy proteins were extracted the same way as the water‐soluble albumin fraction of barley flour (see above) using 0.1 M acetate, pH 5 extraction buffer. The degradation assay with the soy protein extract was done as described for the recombinant rB‐ and rC‐hordein, but with 12 μL soy protein extract as substrate. The samples were run on SDS‐PAGE gel and the relative volume intensities were calculated at the predominant glycinin band (~28 kDa) and two predominant β‐conglycinin bands (~72 kDa) using the Volume tool in the Image Lab 6.1 software (Bio‐Rad). The volume intensities were estimated from bands in an area of 7.7 mm^2^ or 12.1 mm^2^ for Glycinin and β‐conglycinin, respectively. The relative volume intensity (%) was calculated from the adjusted volume intensities of samples, with a pure soy protein sample set to 100% and termed protein level (%).

## Author contributions

MP: performed most of the experiments, data analysis and manuscript preparation. IBH: performed ovule transformation of Stairway and helped with experimental setup and manuscript writing. GD: helped with identifying candidate genes and provided the recombinant hordeins. HB‐P: developed the study concept, grant acquisition, manuscript writing and editing, and supervision.

## Conflict of interest

The authors declare no conflict of interest.

## Supporting information


**Figure S1** Translation of Golden Promise mutant lines.
**Figure S2** CI‐1A and CI‐1B sequence alignments, protospacer comparison and Off‐target sequences.
**Figure S3** CI‐1A Stairway T_0_ and T_1_ mutation sequences.
**Figure S4** Agronomic traits of Stairway ci‐1a mutant and Golden Promise ci‐1a/1b/2 triple mutant.
**Table S1** Immature embryo transformation efficiency of Golden Promise.
**Table S2** Regenerated plants from triple mutant transformation and their zygosity.
**Table S3** sgRNA oligo sequences used for ligation into entry vector pJG85.
**Table S4** Primer sequences and PCR product sizes for target region of all six protease inhibitor genes and hygromycin primers for T‐DNA integration screening.

## Data Availability

The data that supports the findings of this study are available in the supplementary material of this article.
