# Supplementary material for: Simplex and multiplex CRISPR/Cas9‐mediated knockout of grain protease inhibitors in model and commercial barley improves hydrolysis of barley and soy storage proteins
Source: Plant Biotechnol J. 2025 Mar 27;23(6):2418–28. doi: 10.1111/pbi.70065 (PMC12120891; doi:10.1111/pbi.70065)
Supplement: Supplementary file 1 — Figure S1 Translation of Golden Promise mutant lines. Figure S2 CI‐1A and CI‐1B sequence alignments, protospacer comparison and Off‐target sequences. Figure S3 CI‐1A Stairway T0 and T1 mutation sequences. Figure S4 Agronomic traits of Stairway ci‐1a mutant and Golden Promise ci‐1a/1b/2 triple mutant. Table S1 Immature embryo transformation efficiency of Golden Promise. Table S2 Regenerated plants from triple mutant transformation and their zygosity. Table S3 sgRNA oligo sequences used for ligation into entry vector pJG85. Table S4 Primer sequences and PCR product sizes for target region of all six protease inhibitor genes and hygromycin primers for T‐DNA integration screening. [file PBI-23-2418-s001.docx]

# Simplex and multiplex CRISPR/Cas9 mediated knockout of grain protease inhibitors in model and commercial barley improves hydrolysis of barley and soy storage proteins

Michael Panting, Inger B. Holme, Giuseppe Dionisio and Henrik Brinch-Pedersen*

Aarhus university, Department of Agroecology - Crop Genetics and Biotechnology, Forsøgsvej 1, 4200 Slagelse, Danmark

*Correspondence: hbp@agro.au.dk


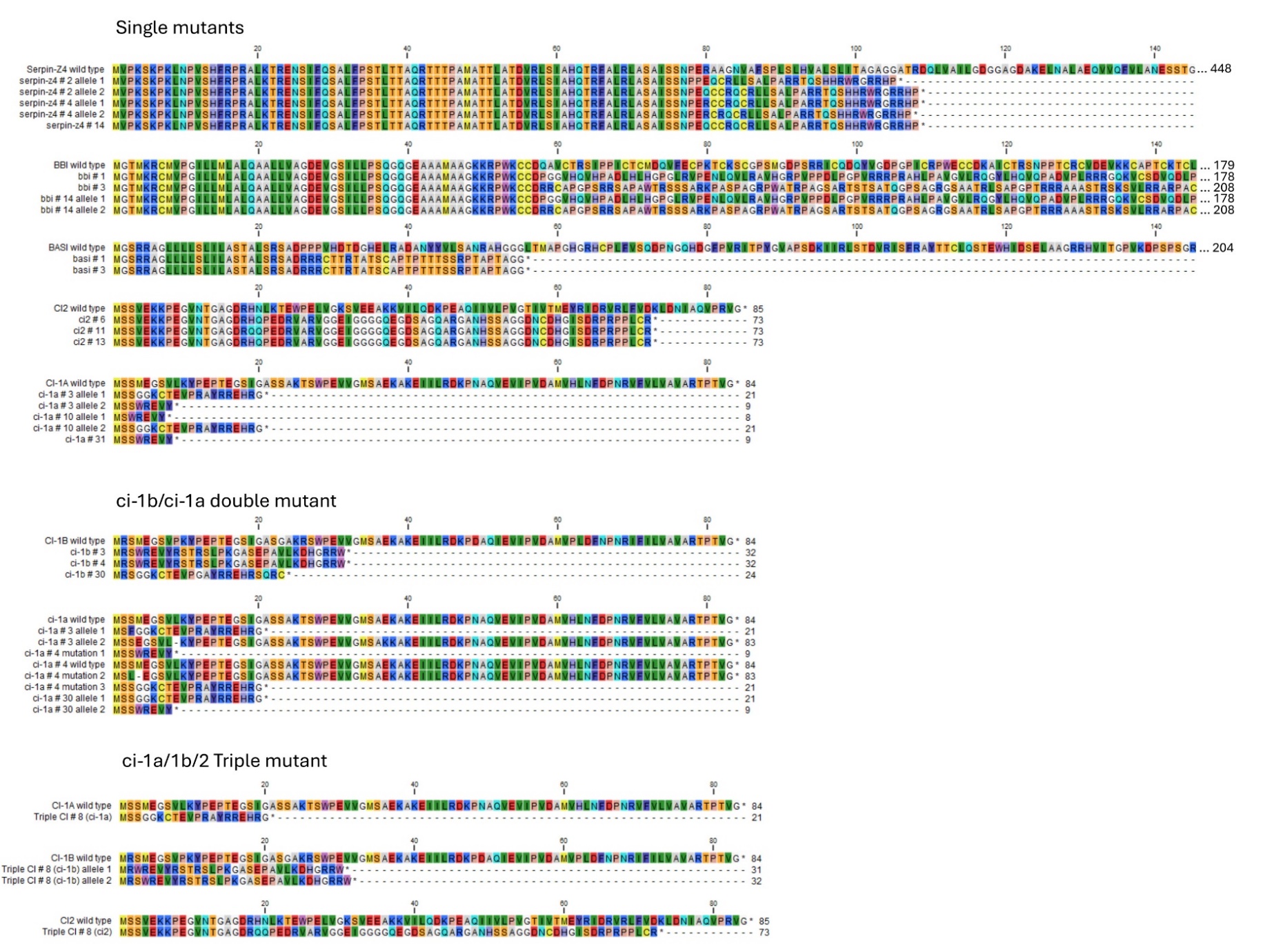
**Supporting Figure S1** Translation of mutant lines used in the study. The heterozygous mutants have two alleles represented and the homozygous mutants have a common translation for both alleles.

**Supporting Figure S2** (A) Sequence alignments of CI-1A and CI-1B genes. (B) Protospacer target sequence of *CI-1A* and *CI-1B*. mismatch at tenth nucleotide from the PAM site in red. (C) Off-target sequences of selected plants. Upper alignment is the CI-1B off-target sequences in the CI-1A knockout plants. Lower alignment is the CI-1A off-target sequences in the CI-1B knockout plants.

**A**
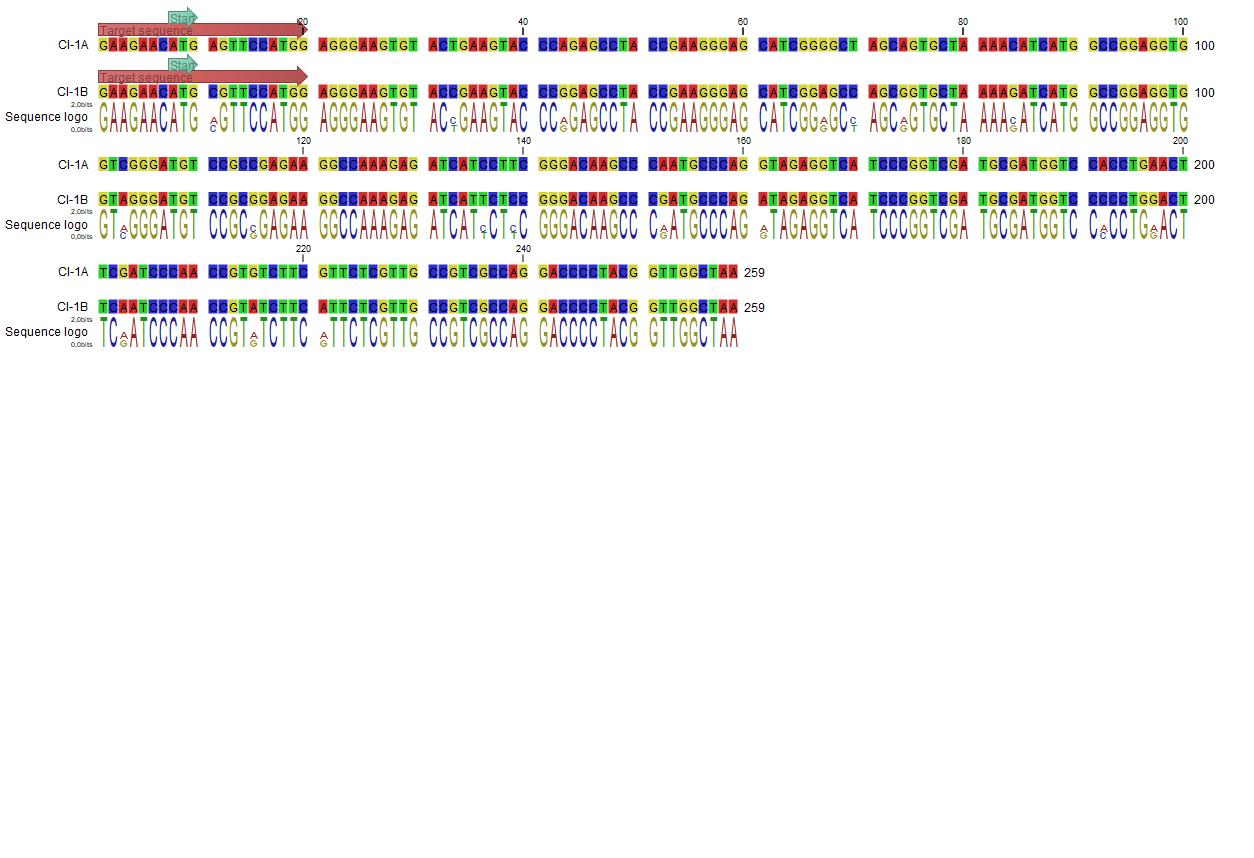


**B**

|  | 5' - 3' sequence |
| --- | --- |
| CI-1A | GAAGAACATGAGTTCCATGGAGG |
| CI-1B | GAAGAACATGCGTTCCATGGAGG |

**C**


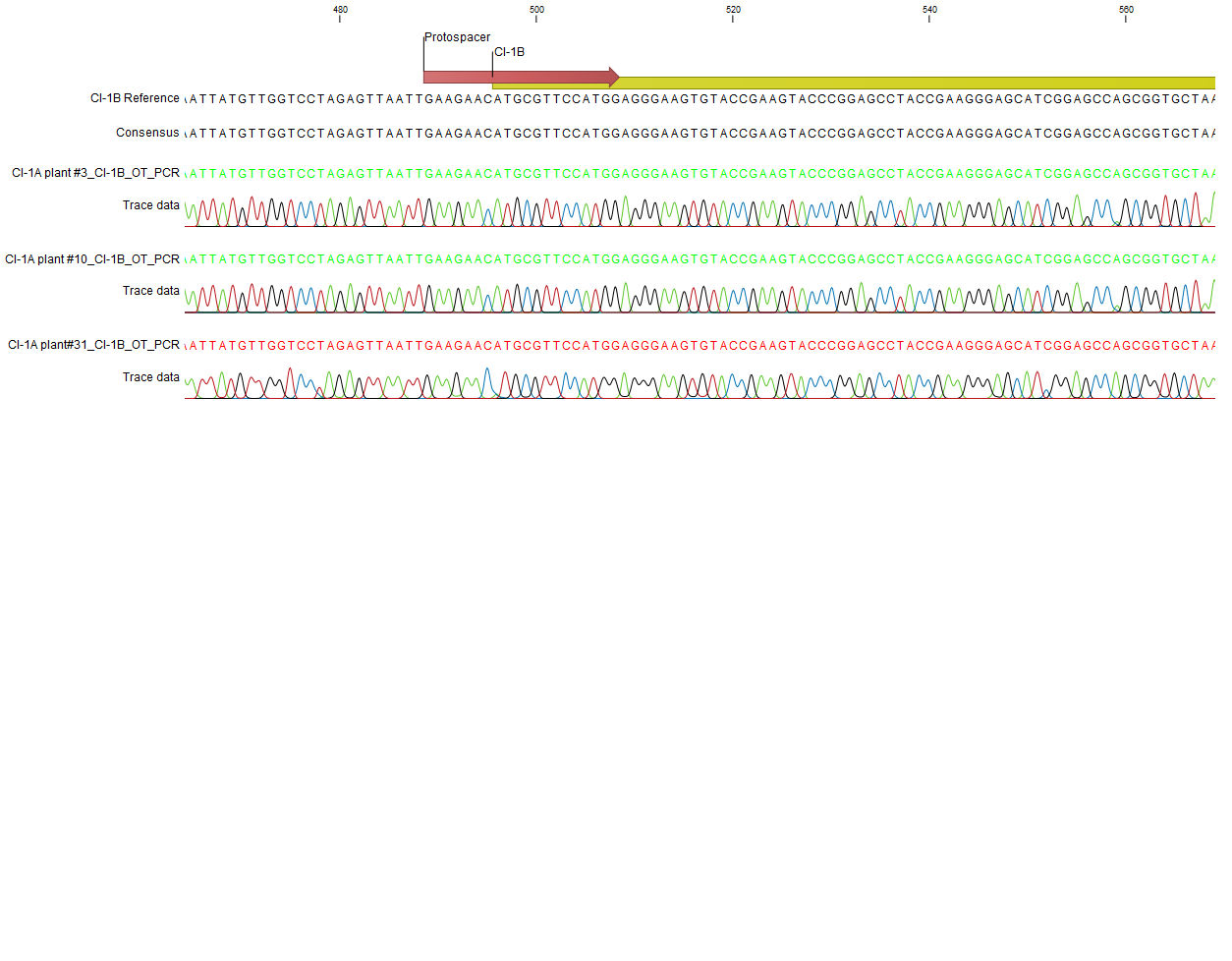


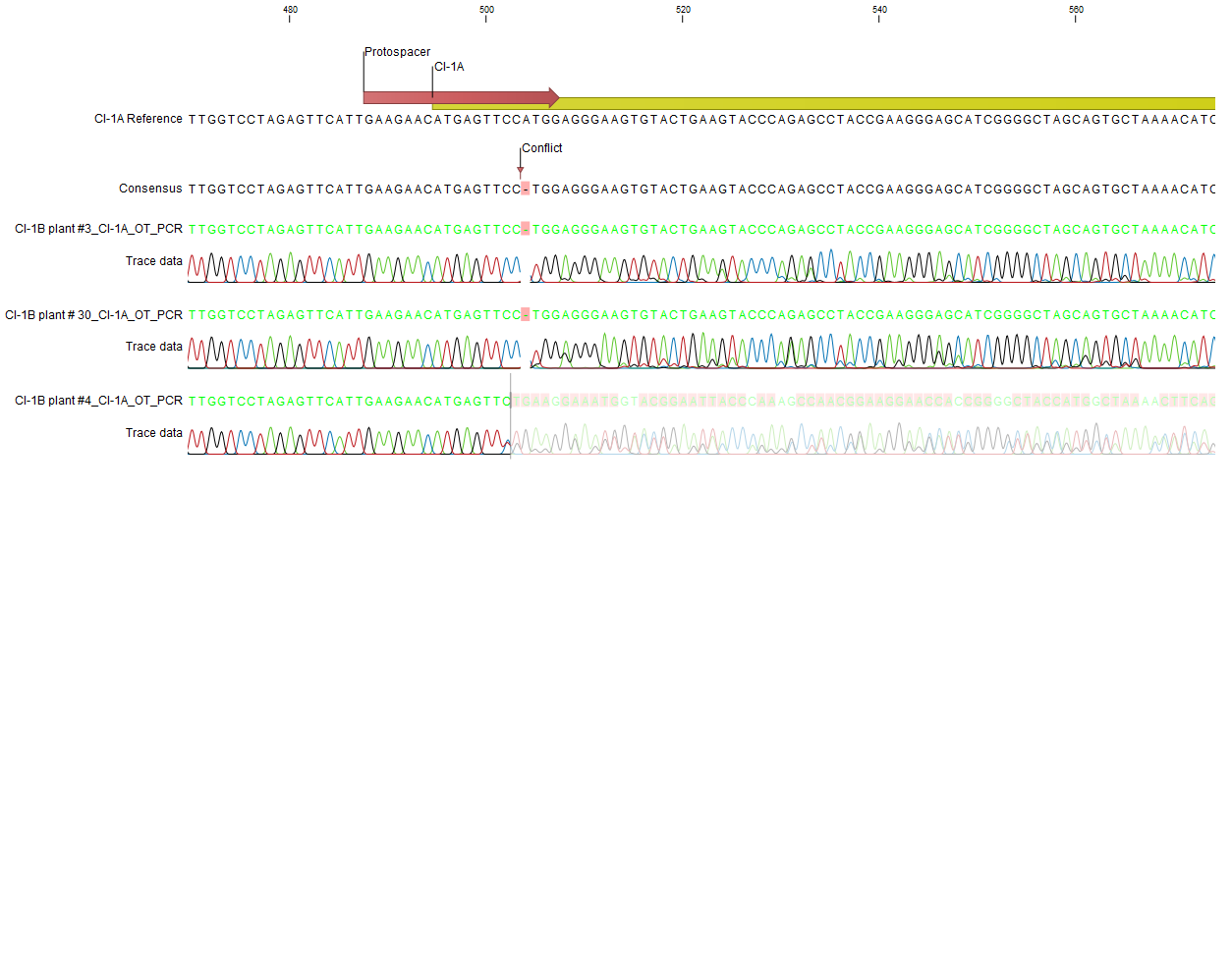


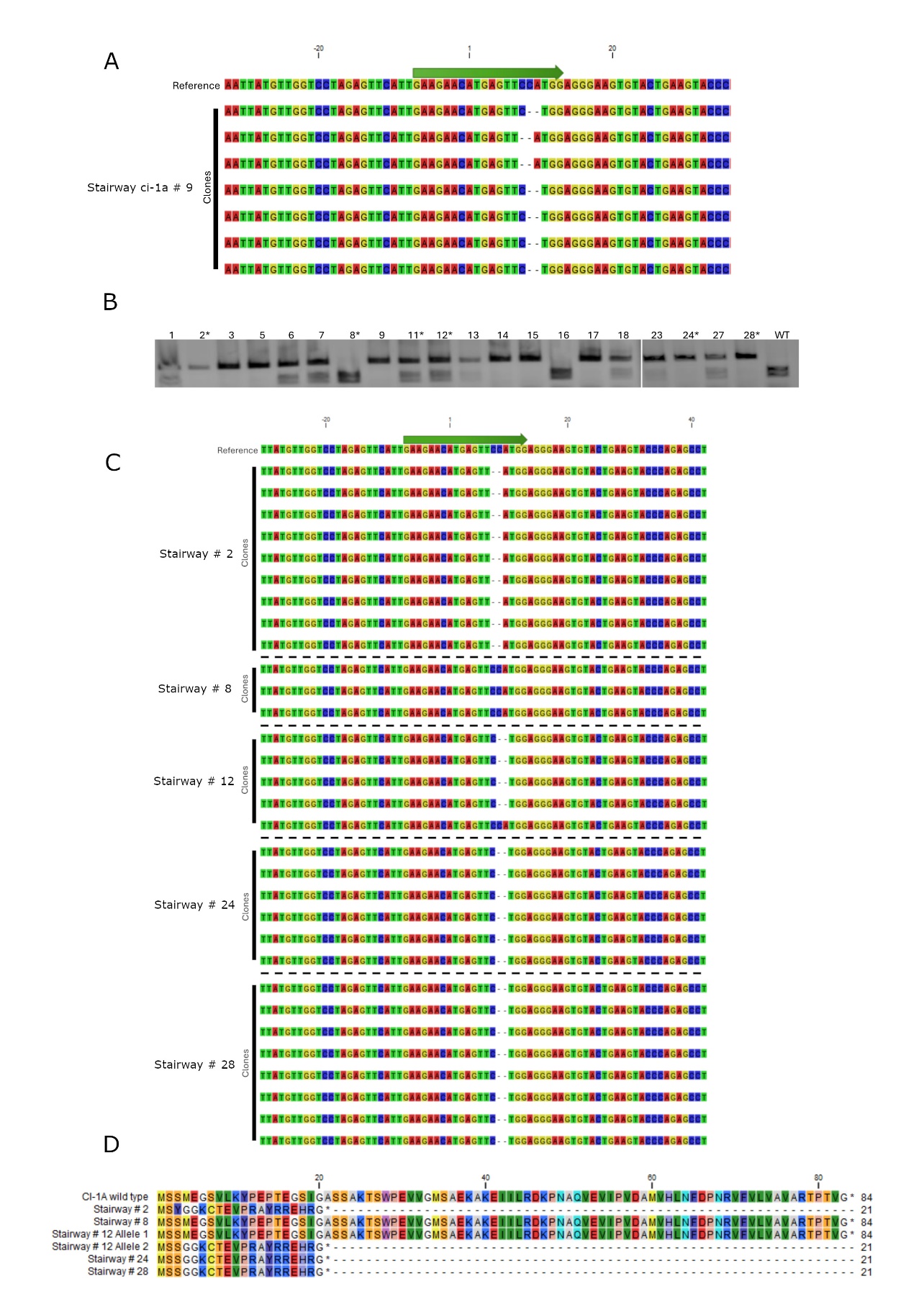
**Supporting Figure S3** *CI-1A* mutation in cv Stairway (A) cloned sequences of chimeric *CI-1A* T_0_ mutant. (B) PCR/RE assay of knock out region in T_1_ progeny using NcoI restriction enzyme. “*” signifies plants used for further analysis. One non-mutated (8); Two Heterozygotic (11 and 12), and three homozygous or biallelic mutants (2, 24,28). (C) cloned sequences of selected T_1_ plants. (D) Amino acid predictions of selected lines.

**Supporting Figure S4** Agronomic traits of Stairway ci-1a mutant and the Golden Promise ci-1a/1b/2 triple mutants grown in greenhouse (T_1_ generation). (A) Table showing average spike number, length and thousand kernel weight (TKW) with standard deviation. No significant differences were observed between wild type and mutants in any of the traits using Student´s t-test. n, number of plants or in the case of spike length, number of spikes measured. (B) Spike pictures. Wt, wild type.

**A**

**A**

| **Plant ID** | **Spikes per plant** | **Spike length (cm)** | **TKW (gram)** |
| --- | --- | --- | --- |
| Stairway wt (n = 3) | 42 ± 1 | 9.53 ± 1.58 (n = 111) | 39.72 ± 4.15 |
| Stairway ci-1a mut (n = 4) | 42.75 ± 10.5 | 9.83 ± 1.57 (n = 157) | 43.61 ± 6.08 |
|  |  |  |  |
| Golden Promise wt (n = 3) | 27.67 ± 9.45 | 9.46 ± 1.08 (n = 35) | 33.72 ± 7.95 |
| ci-1a/1b/2 (n = 3) | 28.33 ± 13.58 | 8.40 ± 1.07 (n = 54) | 31.97 ± 6.36 |

**B**


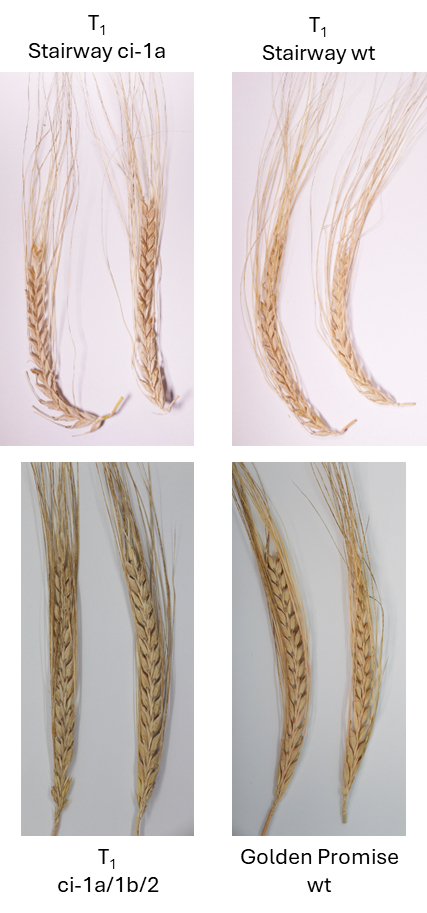


**Supporting Table S1** Immature embryo transformation efficiency of Golden Promise (blue). The zygosity of the regenerated plants were scored, first by PCR/RE and/or PCR sequencing (green). For a few plants, TOPO clones of the PCR products were sequenced to discern if they were heterozygous, bi-allelic or mosaic mutants (red). Wt, wild type. Het, heterozygous. Homo, homozygous.

|  |  |  |  | **PCR sequences** | | | | | | **TOPO clone sequences** | | | |
| --- | --- | --- | --- | --- | --- | --- | --- | --- | --- | --- | --- | --- | --- |
| **Gene Target** | **Transformed embryos** | **Regenerated plants** | **% regenerated** | **Mutated plants** | **Wt** | **% mutants** | **Homo** | **Het** | **% homo** | **Het** | **Bi-allele mutants** | **Mosaic** | **% mosaic** |
| BBI | 75 | 21 | 28.0 | 18 | 3 | 85.7 | 5 | 13 | 27.8 | 0 | 9 | 4 | 30.8 |
| CI-1A | 50 | 36 | 72.0 | 33 | 3 | 91.7 | 2 | 31 | 6.1 |  | 1 |  |  |
| CI-1B/CI-1A^†^ | 50 | 29 | 58.0 | 26 | 3 | 89.7 | 7 | 19 | 26.9 | 1 |  |  |  |
| BASI | 75 | 5 | 6.7 | 2 | 3 | 40.0 | 1 | 2 | 50.0 |  |  |  |  |
| Serpin-Z4 | 75 | 18 | 24.0 | 5 | 13 | 27.8 | 0 | 5 | 0.0 |  | 3 |  |  |
| CI2 | 75 | 13 | 17.3 | 12 | 1 | 92.3 | 0 | 12 | 0.0 |  | 3 | 1 |  |
| † Numbers are based on the primary target CI-1B | | | |  |  |  |  |  |  |  |  |  |  |
|  |  |  |  |  |  |  |  |  |  |  |  |  |  |

**Supporting Table S2** Zygosity of the regenerated plants from the triple mutant generation with mixed agrobacterium (targeting CI-1A + CI-1B + CI2) from immature embryo transformation of Golden Promise. Plant 8 (green) showed mutations in all three genes. Genes *CI-1A* and *CI-1B* were TOPO cloned and sequenced to determine their mutations. Wt, wild type. Het, heterozygous.

|  | | | | | |  |  |  |  |  |
| --- | --- | --- | --- | --- | --- | --- | --- | --- | --- | --- |
| **Plant #** | **1** | **2** | **3** | **4** | **5** | **6** | **7** | **8** | **9** | **10** |
| **PCR** |  |  |  |  |  |  |  |  |  |  |
| CI-1A | wt | wt | het | wt | wt | wt | het | -2 (8) | het | het |
| CI-1B | wt | wt | wt | wt | wt | wt | wt | -4 (5)/-1(3) | N/A | wt |
| CI2 | het | het | wt | het | het | wt | N/A | +1 homo | wt | wt |
| "()" = number of sequenced TOPO clones | | | |  |  |  |  |  |  |  |

**Supporting Table S3** sgRNA oligoes for T4 DNA ligation into pJG85 entry vector. The forward oligo includes 4 bp overhang that is part of the promoter (green) and a G from the scaffold RNA that is excised by Esp3I digestion (red). The reverse oligo includes the 4 bp overhang that is part of the scaffold RNA (red) and a C complementary to the G nucleotide added in the fw oligo.

| **Oligo name** | **Sequence 5'-3'** |
| --- | --- |
| pJG85_**CI-1A**/CI-1B_FW | 5’ACTTGAAGAACATGAGTTCCATGGG |
| pJG85_**CI-1A**/CI-1B_RV | 5'-AAAACCCATGGAACTCATGTTCTTC-3' |
| pJG85_**CI-1B**/CI-1A_FW | 5’ACTTGAAGAACATGCGTTCCATGGG |
| pJG85_**CI-1B**/CI-1A_RV | 5'-AAAACCCATGGAACGCATGTTCTTC-3' |
| pJG85_ **Z4**_FW | 5’ACTTGACATTGCCGGCAGCACGCTG |
| pJG85_ **Z4**_RV | 5'-AAAACAGCGTGCTGCCGGCAATGTC-3' |
| pJG85_**BBI** inh_FW | 5’ACTTGTGGAAGTGCTGCGACCAGGG |
| pJG85_**BBI** inh_RV | 5'-AAAACCCTGGTCGCAGCACTTCCAC-3' |
| pJG85_**CI2**_FW | 5’ACTTGAAGAACATGAGTTCCATGGG |
| pJG85_**CI2**_RV | 5'-AAAACCCATGGAACTCATGTTCTTC-3' |
| pJG85_**BASI**_FW | 5’ACTTGTCGTGCACCGGCGGCGGATG |
| pJG85_**BASI**_RV | 5'-AAAACATCCGCCGCCGGTGCACGAC-3' |

**Supporting Table S4** Primer sequences and product sizes (base pairs)

| **Oligo name** | **Sequence 5'-3'** | **Product size (bp)** |
| --- | --- | --- |
| Hyg fw | ACTCACCGCGACGTCTGTCG | 727 |
| Hyg rv | GCGCGTCTGCTGCTCCATA |  |
| CI-1A fw | CTGTTTTCTTGCTTCCGT | 891 |
| CI-1A rv | CCATGGTATGCTGATGTT |  |
| CI-1B fw | CCTGTTTCGTTGCTTTCCT | 1439 |
| CI-1B rv | GGGTCCTGATGCGTTAATTAT |  |
| CI2 fw | TGTTGAGTTGTATGCAGTGG | 1040 |
| CI2 rv | TGTTTTTTCTTTGGAGGAGTGG |  |
| Serpin Z4 fw | CGTTCTTTTTCTTCCTCTCA | 775 |
| Serpin Z4 rv | GTTCAGTTCCTTGGCGTC |  |
| Bowman-Birk fw | CTGATGGATGATGGTGTGT | 1540 |
| Bowman-Birk rv | GAAGTCGGCAAATCAGGT |  |
| BASI fw | TCCAGCAGAGGTTTCAGT | 698 |
| BASI rv | GGCCAGCCTTATTCAAATGT |  |
